# Triple-Negative Breast Cancer: A Brief Review About Epidemiology, Risk Factors, Signaling Pathways, Treatment and Role of Artificial Intelligence

**DOI:** 10.3389/fmolb.2022.836417

**Published:** 2022-01-25

**Authors:** Nahlah Makki Almansour

**Affiliations:** Department of Biology, College of Science, University of Hafr Al Batin, Hafr Al Batin, Saudi Arabia

**Keywords:** triple negative breast cancer, risk factor, signaling pathways, prognosis, artificial intelligence

## Abstract

Triple-negative breast cancer (TNBC) is a kind of breast cancer that lacks estrogen, progesterone, and human epidermal growth factor receptor 2. This cancer is responsible for more than 15–20% of all breast cancers and is of particular research interest as it is therapeutically challenging mainly because of its low response to therapeutics and highly invasive nature. The non-availability of specific treatment options for TNBC is usually managed by conventional therapy, which often leads to relapse. The focus of this review is to provide up-to-date information related to TNBC epidemiology, risk factors, metastasis, different signaling pathways, and the pathways that can be blocked, immune suppressive cells of the TNBC microenvironment, current and investigation therapies, prognosis, and the role of artificial intelligence in TNBC diagnosis. The data presented in this paper may be helpful for researchers working in the field to obtain general and particular information to advance the understanding of TNBC and provide suitable disease management in the future.

## Introduction

Breast cancer is a pathological condition that occurs in the breast tissue. In most cases, emergence occurs from the milk duct, while other minor cases occur from lobules. The cancer of the ductile region is known as ductal carcinoma, while those involving mammary lobules are called lobular carcinomas (Medina et al., 2020; Muneer et al., 2021). According to the World Health Organization (WHO) reports, breast cancer is placed second on the list of common diseases worldwide. Breast cancer has been observed to cause more mortality in the United States and Europe after lung cancer. The disease is also very common in less developed countries (Ghoncheh et al., 2016; Suleman et al., 2021).

Triple-negative breast cancers or in short TNBCs are regarded as aggressive types of breast cancer and are the product of impaired expression of progesterone and estrogen receptors as well as human growth factor receptor 2 (Bianchini et al., 2016). According to the American Society of Clinical Oncology/College of American Pathologists (ASCO/CAP) guidelines, TNBCs are typically characterized by cellular expression of progesterone and estrogen receptors of ≤1% and human growth factor receptor 2 expressions between 0 and 1+, as determined by immunohistochemistry (Wolff et al., 2014). There are four transcriptional subtypes of TNBCs: two basal subtypes, which are grouped as BL1 and BL2, a mesenchymal subtype M, and a luminal androgen receptor subtype (Lehmann et al., 2016). Further, TNBC can be categorized into six different subgroups based on their molecular heterogeneity: immunomodulatory, luminal androgen receptor expression, mesenchymal stem-like, mesenchymal-like, basal-like, and unstable (Yam et al., 2017). TNBCs constitute 12–17% of all breast cancers and are naturally recurrent. Scientifically, this cancer is categorized as a distant subgroup within a broad category of breast cancers (Foulkes et al., 2010). The clinical behavior of TNBCs is relatively aggressive compared to that of other subtypes of breast cancer. Additionally, these cancers have characteristic metastatic patterns and poor prognosis (Dent et al., 2007). TNBCs represent 24% of newly diagnosed breast cancers, and a steady increase has been reported in their incidence (Tsai et al., 2016). It has been reported that in 2018 about 2,088,849 cases of TNBC were reported making it a common cancer in women (Singh et al., 2020). The average survival rate from the disease is ∼10.2 months in perspective on the currently available therapy, with a 65% 5 years survival rate in cases of regional tumors and 11% for those where the tumor is spread to distant organs (Kohler et al., 2015).

In this review, the focus is to provide a comprehensive overview of TNBCs, epidemiology and risk factors, signaling pathways, prognosis, current and investigational treatment paradigm, and the role of artificial intelligence in TNBC diagnosis and treatment.

## Epidemiology of TNBC

TNBC accounts for 15–25% of all breast cancers (Yin et al., 2020). The TNBC proportion in all age groups followed a similar trend (Hudis and Gianni, 2011; Khan et al., 2017). However, younger and older women have increased rates of BRCA and basal TNBC and apocrine and neuroendocrine TNBC. African American and Hispanic women are found to be at high risk of TNBC, and African Americans have a worse prognosis compared to other groups (Reynolds, 2007). In a case study conducted in 2009, 187 TNBC patients were reported to have a 2.5% higher risk for TNBC who used oral contraceptives for more than 1 year. The risk is 4.2% among women aged less than 40 years. It was also noted that when the duration of oral contraceptive use increased, the risk increased. In the United States, TNBC is responsible for 12% of breast cancers, with a 5-years survival rate of 8–16% (Howard and Olopade, 2021).

## Potential Risk Factors

The potential risk factors of TNBC can be divided into non-modifiable and modifiable risk factors.

## Non-Modifiable Risk Factors

### Age

Approximately 80% of breast cancers (including TNBCs) are >50 years old (Donepudi et al., 2014). The cancer risk increases with age as follows: 1.5% risk at the age of 40 years, 3% at age 50, and more than 4% at age 70 years (Łukasiewicz et al., 2021). In addition, a relationship exists between cancer subtype and age. This can be explained by TNBC, which is mostly diagnosed in the age group of <40 years, whereas in patients aged >70 years, luminal A subtype cancer is more common (McGuire et al., 2015).

### Sex

Due to different sex hormonal stimulation, female sex is considered a higher risk for TNBC compared to male sex. Females have breast cells that are very susceptible to estrogen and progesterone hormones, as well as imbalances. Circulation of estrogens and androgens is associated with an increased risk of breast cancer (Hormones et al., 2013). In the case of premenopausal and postmenopausal women, physiological changes in endogenous sex hormones result in a higher risk of breast cancer (Hormones and Group, 2002; Zhang et al., 2013). In men, the prevalence of breast cancer is 1%. The important factors which increase a man’s risk of breast cancer are; older age, “BRCA2/BRCA1” mutations, and increased estrogen levels, genetic history in family, and highly exposure to radiation (Giordano, 2018).

### Genetic Mutations

Mutations in genes such *as BRCA1* and *BRCA2* were found to be strongly associated with TNBC (Shiovitz and Korde, 2015). Mutations in *TP53*, *CDH1*, *PTEN*, and *STK11* are also associated with breast cancer and TNBC incidence (Corso et al., 2018; Shahbandi et al., 2020). Mutations in the *XRCC2* gene are also associated with high risk of breast cancer (Kluźniak et al., 2019). Further, it has been revealed that *BRCA1*-related tumors profile resembles the TNBC subtype, while the profile of the BRCA2-associated tumor correlates to luminal-like breast cancers, particularly the Luminal B subtype (Incorvaia et al., 2020).

### Race/Ethnicity

The incidence of TNBC remains high among white non-Hispanic women (Hill et al., 2019). In addition, the mortality rate is significantly higher among black women, and black women are considered to have the lowest survival rates for malignancy (DeSantis et al., 2016).

### Genetic History

Genetic history is one of the major risk factors associated with breast cancer (similar to TNBC). Approximately 13–19% of diagnosed breast cancer patients report a first-degree breast cancer relative (Cuzick, 2003). Moreover, the risk is higher in family members of age <50 years (Baglia et al., 2018). The genetic history of ovarian cancer in a family, particularly those with *BRCA1* and *BRCA2* mutations, has a greater risk (Wu et al., 2018).

### Breast Tissue Density

As per clinical practice, breast tissue density has been categorized as low-density breasts, fatty, and high-density breasts. Women receiving hormone replacement therapy are reported to have denser breasts during early age, during pregnancy, and breastfeeding, even with lower BMI (Titus-Ernstoff et al., 1998). In postmenopausal and premenopausal women, the density of the breast affects the risk of cancer, that is, the higher the density, the higher the chances (Checka et al., 2012). Breast tissue density screening could be a promising and quick approach for the rational surveillance (Kim et al., 2020).

### History of Radiation Therapy

A history of radiotherapy can lead to the development of secondary tumors. This is mainly dependent on the patient’s state and age (Ng and Shuryak, 2015). Patients aged <30 years are considered at higher risk (Zhang et al., 2020), and radiotherapy treatments, such as multiple-field IMRT (6F-IMRT) and double partial arc (VMAT) techniques can increase the chances of secondary tumors (Balaji et al., 2016). Radiotherapy in patients with a family history of breast cancer is considered to be at a higher risk (Bartelink et al., 2001).

### History of Breast Diseases

The initial symptoms of cancer are cancerous lesions in the breast (Schacht et al., 2014; Muneer et al., 2019). Regarding the family history of disease, the other risk factors associated with breast cancer are; *in-situ* carcinoma, atypical hyperplasia, proliferative lesions and non-proliferative lesions (Dyrstad et al., 2015; Socolov et al., 2015). Breast cancer risks include a family history of breast cancer and benign lesions (Wang et al., 2004).

## Modifiable Risk Factors

### Drugs

Diethylstilbestrol is a major cause of breast cancer during pregnancy (Hoover et al., 2011). Although much more study and research is required to support this statement, diethylstilbestrol intake and consumption by pregnant women not only causes breast cancer in the mother but also the child (Hilakivi-Clarke, 2014). This relationship is observed with diethylstilbestrol uptake even without the expression of estrogen and progesterone receptors (Palmer et al., 2006). The breast cancer risk increases with an increase in diethylstilbestrol doses. Female age is another consideration, that is, the risk increases 1.9 times in women older than 40 years (Narod, 2011). Hormonal replacement therapy, when carried out for more than 5–7 years, increases the chances of breast cancer. The continuous uptake of the selective antidepressants, paroxetine, tricyclic, and serotonin inhibitors, also increases the chances of breast cancer (Cotterchio et al., 2000). Similarly, Friedman reported tetracycline can increase risk of breast cancer (Friedman et al., 2006). Furthermore, the relationship between the risk of breast cancer and excessive use of hypertensive medications, anti-inflammatory non-steroidal drugs, and statins has also been studied, but the research data in this regard are not efficient in supporting these data (Coogan et al., 1999; Denoyelle et al., 2001).

### Body Mass Index

According to several epidemiological studies, obesity is a potential risk factor for breast cancer. Epidemiologically, estrogen receptor-positive breast cancer develops in obese women in the postmenopausal period (Kolb and Zhang, 2020). However, women more than 50 years of age with greater BMI are at higher risk of breast cancer than those with low BMI (Wang et al., 2019). However, it has been reported that people with a higher BMI are at a high risk of tumors with a high percentage and size of lymph node metastasis. In premenopausal women, obesity is not only an evident cause of cancer, but also high mortality (James et al., 2015). Procarcinogenic events are facilitated by greater fat content in the body, which in turn enhances the circulation of hormones and inflammation. Females with a BMI greater than 25 kg/m^2^ had poor clinical outcomes (Protani et al., 2010). Greater fat contents, although with the relevant BMI in post-menopausal women, have poor clinical outcomes. People with a family history of breast cancer are at a greater risk of breast cancer with greater BMI (Iyengar et al., 2019).

### Physical Activity

The physical activity is considered the best action to be performed in order to prevent breast cancer (Kyu et al., 2016). This is supported by the study of Chen et al. that in women the breast cancer occurrence is reduced by physical activity during the postmenopausal period (Hormones et al., 2013), The Physical activity reduces the exposure to endogenous sex hormones and can also alter insulin-like growth factor-1 levels and immune responses (Hoffman-Goetz et al., 1998; Hormones et al., 2013).

### Alcohol Intake

Various studies reported alcohol consumption is a major cause of cancer in the gastrointestinal tract, along with breast cancer (Rachdaoui and Sarkar, 2013). Alcohol and alcohol beverages can increase the risk of malignancy. The hormone balance is disturbed along with the enhanced production of estrogen, which in turn increases body weight. Alcohol and its beverages are considered to increase the risk of cancer growth (Coronado et al., 2011). Alcohols are the major causative agents of estrogen-positive breast cancers (Zeinomar et al., 2019). Morphological alterations of the breast and its tissues have been reported with the consumption of alcohol before the 1st pregnancy (Liu et al., 2015).

### Insufficient Vitamin Supplements

Vitamins are anti-cancer elements that can prevent breast malignancies. Research is underway to evaluate the risk of cancer with the consumption of vitamins, particularly vitamin B, C, and E folic acids and multivitamins (Cui and Rohan, 2006). Vitamin D supplements, that is, high serum 25-hydroxyvitamin D, are thought to be potential cancer control agents in postmenopausal women and in the premenopausal period (Atoum and Alzoughool, 2017). Excessive expression of vitamin D receptors is associated with a lower mortality rate in patients with breast cancer (Zhou et al., 2020). Artificial light exposure for a longer duration can increase the risk of breast malignancy (Al-Naggar and Anil, 2016). This occurs because of the activation of melatonin pigments and consequent epigenetic shifts (Johns et al., 2018).

### Exposure to Chemicals and Drugs

Females who have been exposed to dreadful carcinogenic chemicals are at higher risk of breast cancer and epigenetic alterations and mutations. Exposure and duration of exposure contribute to an increased risk of breast cancer mutagenesis (Casey et al., 2015). Exposure of mammary glands to polychlorinated biphenyl (PCB) and dichlorodiphenyltrichloroethane (DDT) chemicals increases the risk of breast cancer (Leso et al., 2019). Furthermore, continuous exposure to organic solvents, insecticides, and oil mist increases the risk of breast cancer (Videnros et al., 2020). Antibiotics, statins, antidepressants, and antihypertensive drugs can increase the risk of breast cancer. Similarly, NSAIDs that contain aspirin and ibuprofen are considered major risk factors for breast cancer (Brandes et al., 1992; Bjarnadottir et al., 2013).

### Smoking

Tobacco causes mutations in oncogenes and p53 suppressor genes (Terry and Rohan, 2002). Active smoking passive smokers are at a risk of cancer. Smoking during pregnancy and chain smokers are at potential risk of malignancies (Couch et al., 2001).

### Intake of Processed Food/Diet

According to the WHO, processed foods, such as meat, are confirmed group-1 carcinogen for gastrointestinal cancer and breast malignancy (Dandamudi et al., 2018). The excessive use of saturated fats is also considered a carcinogen. The obesity-causing ultra-processed diet plans that are enriched in elements such as sugar, sodium, and fats are thought to be carcinogenic and increase the risk by 11% (Fiolet et al., 2018). Diets that are rich in green vegetables, fresh fruits, protein-enriched grains, and legumes are anti-carcinogenic and therefore reduce the risk of breast cancer (Castello et al., 2014). Similarly, diets rich in phyto-estrogen, folate elements, saturated fibers, n-3 PUFA, and vitamin D are regarded as anti-cancer agents (Dunneram et al., 2019). Hence, a low dose consumption of saturated fat and n-6 PUFA has been proposed (Li et al., 2014). The antioxidants found in green tea have also shown anti-carcinogenic properties (Liu and Chen, 2013). Curcuminoids and sulforaphane (SFN) derived from turmeric are thought to be anti-carcinogens (E Wright et al., 2013).

### Complexities of TNBC Metastasis

TNBC is one of the most aggressive subtypes of cancer that is often associated with poor patient outcomes because of the development of metastases in secondary organisms like in the brain, bone, and lungs (Zeichner et al., 2016). Metastatic growth to these distant organs, represents a significant clinical challenge, as metastatic disease is currently incurable and is a primary death cause for the vast majority of TNBC patients. Metastatic spread of cancer is a complex, poorly understood process, and involves multiple steps, such as angiogenesis acquisition of invasive properties through epigenetic and genetic alterations, intravasation through the basement membrane, extravasation of some cancer cells to distal tissues, and tumor-stroma interactions (Nguyen and Massagué, 2007; Fatima et al., 2018). Metastatic cells outgrowth in a foreign tissue environment is considered as the rate-limiting step of breast cancer metastasis and in this stage, breast cancer cells are difficult to detect and show resistance to chemotherapy due to lack of proliferation (Giancotti, 2013; Dujon et al., 2021). To date, this remains a clinical obstacle, since the patients considered as “survivors” can develop metastatic tumors years later. Disseminated tumor cells can enter a state of dormancy in the secondary organs by achieving a balanced state of proliferation and apoptosis. Successful emergence from dormancy is the result of further evolution of surviving disseminated tumor cells by accumulating molecular changes as well as *via* permissive interactions with the tumor microenvironment (Giancotti, 2013). By achieving these characteristics, metastatic tumor cells can optimally adapt to the host microenvironment and initiate colonization. While significant information has been generated to identify the specific processes required for breast cancer initiation, still, much effort to elucidate the molecular mechanisms and roles of critical genes and signaling pathways involved in the late stages of metastatic growth are required.

## Signaling Pathways

### Notch Signaling Pathway

**FIGURE 1:**
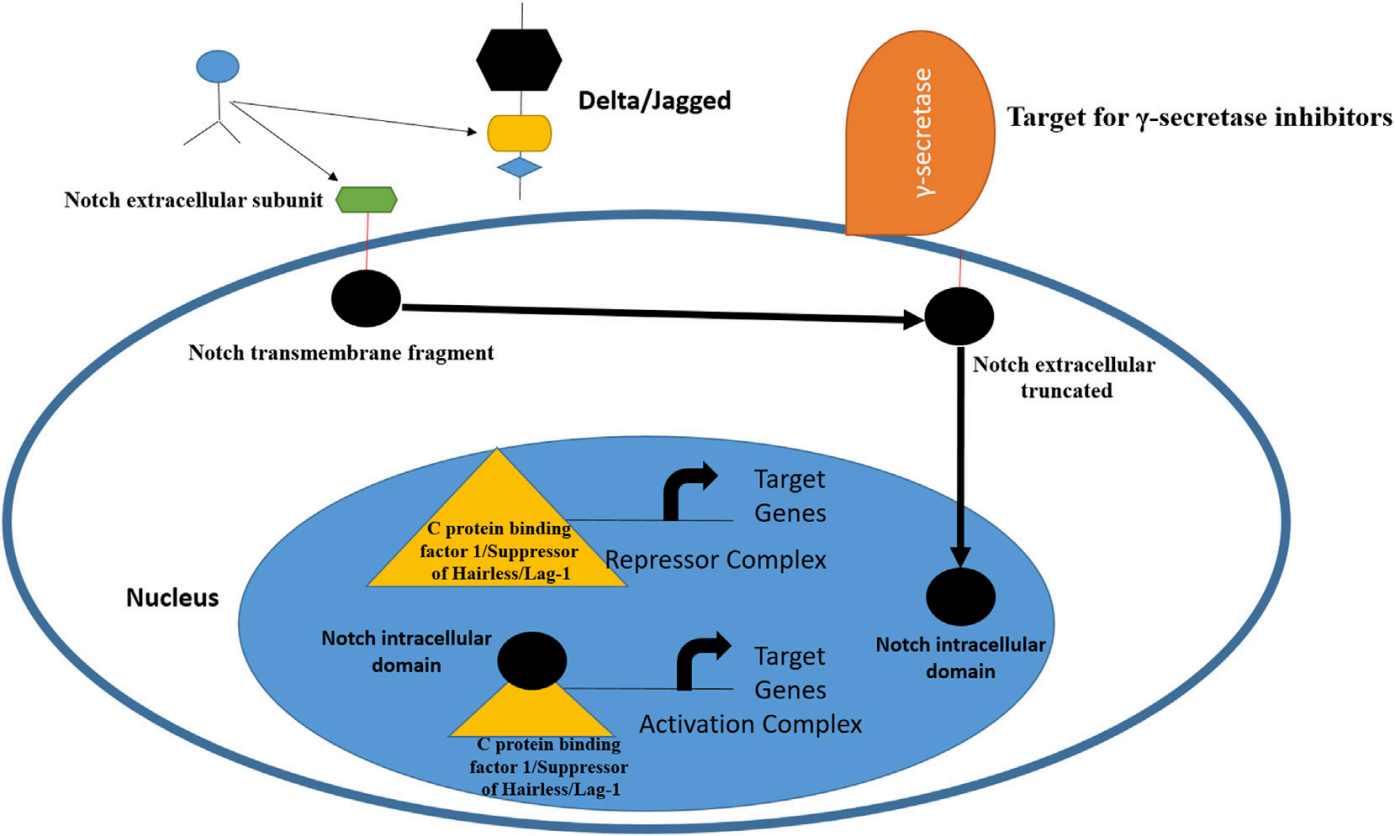
Schematic representation of Notch receptor activation and avenues for therapeutic intervention. The pathway is activated by ligand binding to Notch receptor, followed by proteolytic cleavage fy proteases. This releases the Notch intracellular domain, which is then transferred to nucleus for biding with C protein binding factor 1/Suppressor of Hairless/Lag-1 allowing conversion of complex from repressor to activator of Notch genes. From inhibition perspective, γ-secretase inhibitors and monoclonal antibodies can inhibit Notch ligands and receptors (Yuan et al., 2015; Medina et al., 2020).

The term Notch was first described by Thomas Hunt Morgan in 1917 and refers to transmembrane receptors and ligands (Gu et al., 2016; Fatima et al., 2018). This juxtacrine signaling pathway plays a central role in the developmental process and uses communication among cells *via* transmembrane interactions with ligands (Yaron et al., 2014). The Notch pathway is a short-range cell-to-cell communication pathway that is critical for metazoan development (Artavanis-Tsakonas et al., 1999). The Notch receptor is expressed on the plasma membrane and cleaved by furin-like convertase in the Golgi compartment (Blaumueller et al., 1997). The Notch pathway has been identified in *Drosophila melanogaster.* From a structural perspective, Notch receptors share three domains: an intracellular domain, a transmembrane region, and an extracellular domain (Bellavia et al., 2008). This signaling pathway is key in cell proliferation and differentiation (Palomero et al., 2006) most importantly, governs embryonic development and maintains tumor stemness to TNBC tumor metastasis. There are four Notch receptors and five ligands in this pathway. The receptors can be named as Notch 1 to 4, while ligands are Delta-like 1, Delta-like 3, Delta-like 4, Jagged-1, and Jagged-2 (Speiser et al., 2013). Several reports have indicated the overexpression of Jagged 1 and Delta 1 in breast cancer. The Notch 1 is involved in pancreatic cancer and hematological malignancies, while Notch-3/4 has been found to assist in tumor proliferation and survival. Notch-2 overexpression in TNBC appears to play a protective role (Weijzen et al., 2002). Notch expression has been linked to TNBCs, and scientists believe that targeting receptors by monoclonal antibodies can reduce HES and HEY-L families (Sharma et al., 2012). The monoclonal antibody based on the delta-like ligand 4 Notch ligand also showed effectiveness in treating TNBC (Benedito et al., 2009). Inhibitors that target the Notch signaling pathway act at proteolytic cleavage and block the formation of multimeric γ-secretase complexes; thus, these drugs are termed γ-secretase inhibitors (Chan et al., 2007). The Notch pathway and the points at which it can be blocked are shown in Figure 1.

### Wnt/β-Catenin Pathway

Different Wnt ligands, such as WNT3A, WNT11, and WNT5A, are reported to be pertinent in cancer migration and invasion (Zhu et al., 2012). In particular, the FZD6 receptor is associated with increased malignant cell motility in TNBC (Corda et al., 2017). It has been revealed that OMP-18R5 antibody targets Frizzled receptors to diminish tumor cell proliferation in colon, lung, breast, and pancreatic tumors (Gurney et al., 2012). Wnt/β-catenin signaling pathway is activated in epithelial ovarian cancer and targets gene regulate cell proliferation and apoptosis thereby mediating cancer initiation and progression. Furthermore, Wnt inhibitors can destroy drug-resistant cells and cancer stem cells (Dean et al., 2005).

### TGF-β Signaling Pathway

TGF-beta 1 is expressed exponentially in TGF-β1 and TGF-β1 and has been implicated in its important role in breast cancer stem cells (Jamdade et al., 2015). *In vivo* analysis, inhibition of TGF-β leads results in multiplication and growth of tumor cells. The frequent overexpression of TGF-β in the TNBC tumor microenvironment, particularly in stromal, tumor-associated immune cells, and tumor cells. In these cells, SMAD4 and SMAD2/3 cause metastasis and angiogenesis (Bhola et al., 2013). Thus, inhibition of TGF-β plays a significant therapeutic role in patients with metastasis.

### Signaling Pathway of CSPG4 Protein

The CSPG4 protein (non-glial antigen) is expressed as a cell surface proteoglycan by basal breast carcinoma cells. Therapeutically, CSPG4 inhibition allows for efficient management of breast cancer (Wang et al., 2010). Monoclonal antibodies can block the CSPG4 protein, which hinders survival signaling pathways in tumor cells. In addition, controlling the overexpression of CSPG4 by targeting it therapeutically is seen in different TNBC cells (Cooney et al., 2011).

### Hedgehog Signaling Pathway

The Hedgehog signaling pathway is involved in cancer cell invasion, metastasis, drug resistance, and tumor recurrence (Li et al., 2012). Overexpression of this pathway results in poor prediction of breast cancer mortality, especially in TNBC patients. The Hedgehog signaling pathway is considered to initiate breast cancer malignancy. Thiostrepton is a novel experimental drug that suppresses TNBC CD44^+^/CD24^−^ cancer stem cells (Yang et al., 2016).

### PI3K/AKT/mTOR Pathway

Rapamycin and paclitaxel drugs are used to inhibit the PI3K/AKT/mTOR pathway and hence play a significant role in TNBC treatment. Furthermore, mTOR antibodies are considered more effective than mTOR inhibitors alone (Ali and Wendt, 2017). In TNBC patients, ipatasertip (an AKT inhibitor) can promote progression-free survival by inactivating the PI3K/AKT pathway. Despite these efforts on PI3K/AKT/mTOR pathway inhibitors, synthesis of novel inhibitors is needed to block the PI3K/Akt/mTOR pathway and act as therapeutic agents against TNBC (Blanco et al., 2014).

### Epidermal Growth Factor Receptor

**FIGURE 2:**
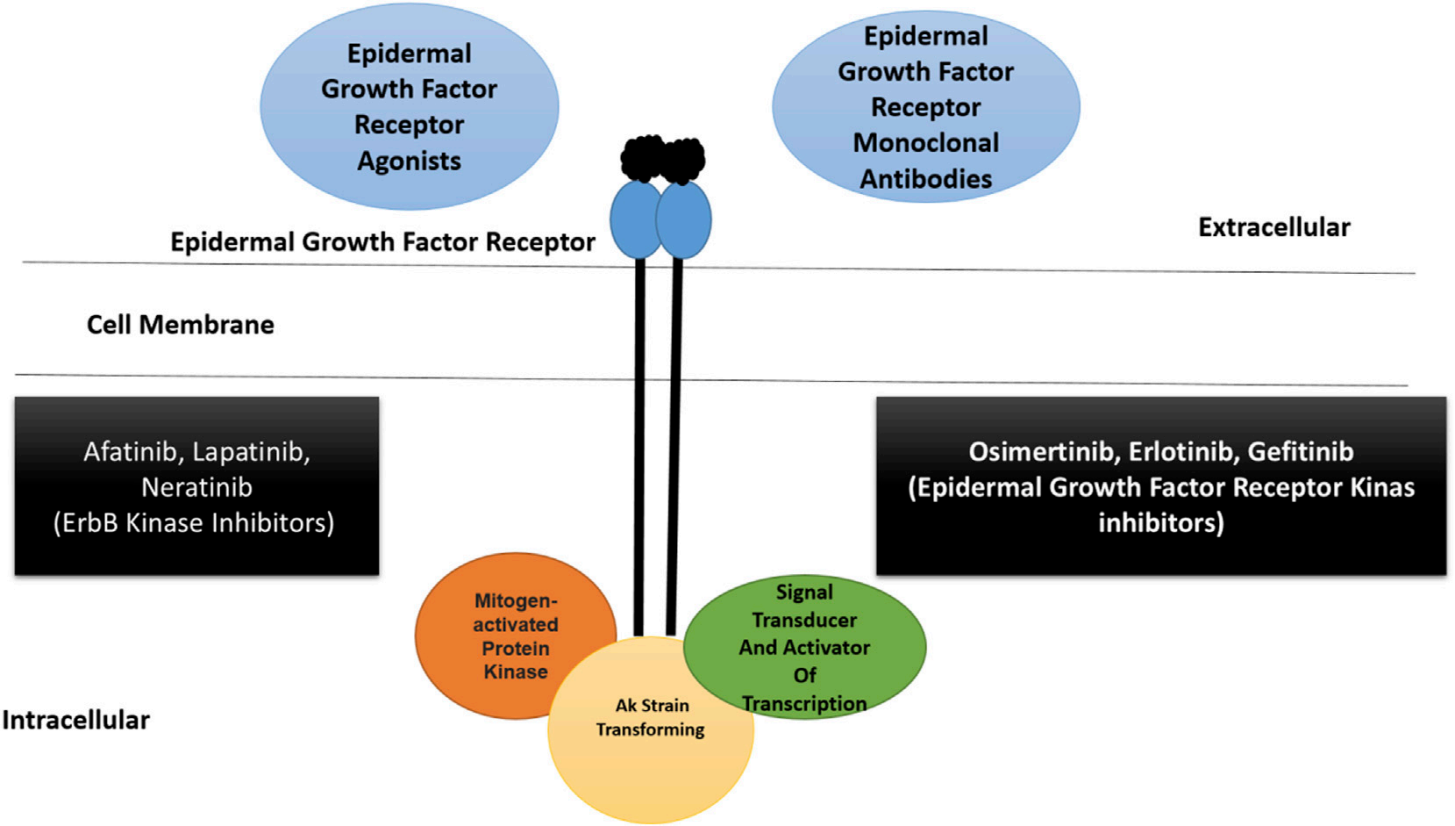
A schematic illustration of epidermal growth factor receptor signaling pathway along with activators and inhibitors. The pathway is part of ErbB superfamily. The epidermal growth factor receptor is able to bind to different ligands at the extracellular surface resulting in the activation of downstream signaling events. Therapeutics including monoclonal antibodies and different kinase inhibitors can block the binding of ligand to the receptor. The kinase inhibitors can also block the function of other ErbB receptors (Ali and Wendt, 2017; Medina et al., 2020). Inhibitors of polyadenosine diphosphate-ribose polymerase.

The epidermal growth factor receptor is reported in 89% of TNBC and is considered an attractive therapeutic target, particularly in BL2 subtype tumors (Sobande et al., 2015). The expression of this gene results in primary tumorigenesis and metastasis. The EGFR inhibitor gefitinib lowers the proliferation of cancer cells and increases carboplatin and docetaxel cytotoxicity (Eccles, 2011). Several EGFR inhibitors, such as lapatinib and erlotinib, are currently being tested against TNBC, in addition to cetuximab and panitumumab (monoclonal antibodies) (Nabholtz et al., 2014; Hsiao et al., 2015). The synergistic therapeutic approach of monoclonal antibodies and chemotherapeutics is considered to be more effective. This can be exemplified by the combined use of carboplatin and cetuximab, and cisplatin and cetuximab proved to be more efficacious in patients with advanced TNBC (Carey et al., 2012). Additionally, tri-inhibitor therapy, including carboplatin, gefitinib, and docetaxel, enhances TNBC cytotoxicity. Cannabidiol inhibits breast cancer metastasis by interfering with the epidermal growth factor pathway (Velasco et al., 2016). The epidermal growth factor receptor signaling pathway is presented along with activator and inhibitor points of action, as shown in Figure 2.

**FIGURE 3:**
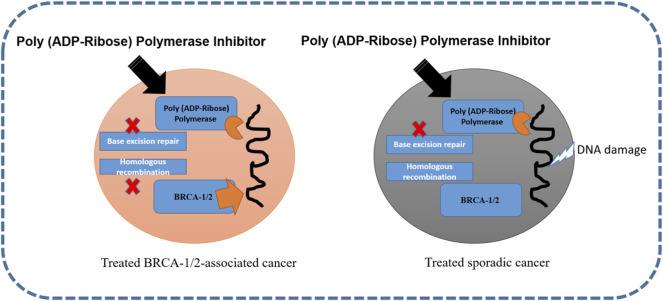
Inhibition of poly (ADP-ribose) polymerase in BRCA-1/2-associated and sporadic cancers (Ellisen, 2011; Medina et al., 2020).

Polyadenosine diphosphate-ribose polymerase is involved in molecular mechanisms that allow cells to recover from DNA damage, apoptosis, gene transcription, and genomic stability (Park and Chen, 2012). In approximately 70 and 23% of breast cancers, BRCA1 and BRCA2 mutations have been reported (Mahfoudh et al., 2019). For both these mutations and TNBC, PARP inhibitors are regarded as the most vital drugs. The activation of PARP-1 and PARP-2 proteins is a consequence of DNA strand breaks. The polyadenosine diphosphate-ribose polymerase enzyme is synthesized by PARP and drives the single-strand break repair and excision repair pathway (De Vos et al., 2012). It has been observed that when PARP activity is affected, it blocks DNA polymerase ε for DNA damage repair (Helleday, 2011). Veliparib and olaparib are both PARP inhibitors that have different catalytic inhibition mechanisms (Murai et al., 2012). The inhibition of poly (ADP-ribose) polymerase in BRCA-1/2-associated and sporadic cancers is shown in Figure 3.

### Mammalian Target of Rapamycin

**FIGURE 4:**
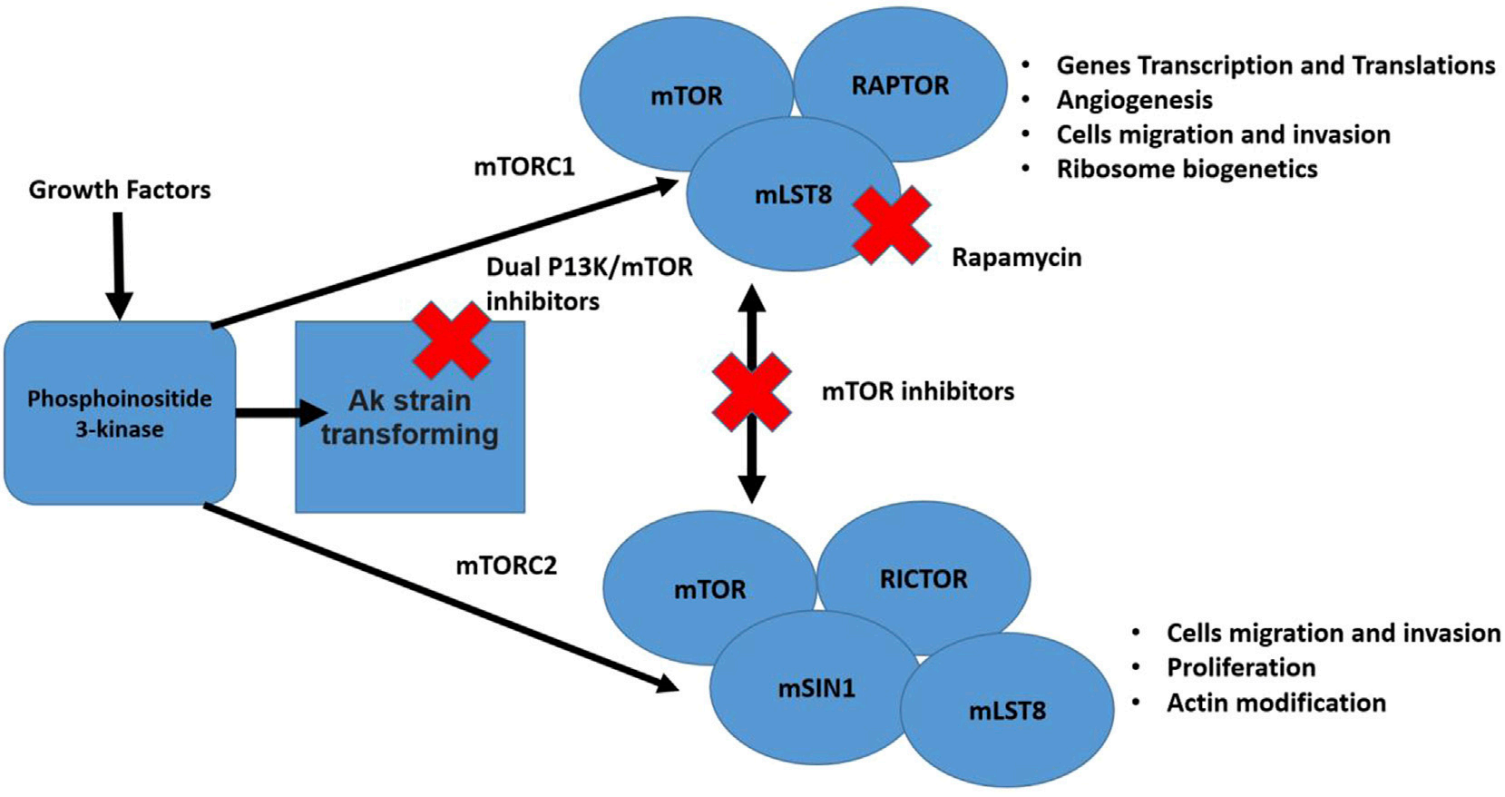
The mTor pathway illustrating two distinct complexes (mTORC1 and mTORC2). The pathway is stimulated by different growth factors. The mTORC2 activated Akt. Multiple cell functions are regulated by both mentioned complexes that are considered vital for cancer development. Also, in the figure different steps of the pathway that can be blocked by inhibitors are shown (Xie et al., 2016; Medina et al., 2020).

The mTor pathway is responsible for poor prognosis due to the aggressive nature of the cancer and its good tissue invasion property (Xie et al., 2016). Errors in the mTOR pathway are strongly correlated with malignancy (Fruman and Rommel, 2014). The phosphorylation reactions of this pathway are also associated with proliferation, vascular endothelial growth factor, and angiogenesis, that enhance endothelial cell growth (Arcaro and Guerreiro, 2007). Moreover, high expression of a protein kinase enzyme (Akt) has been reported to be involved in tumor invasion and metastasis therefore, inhibiting the mTOR pathway can be an efficient anti-cancer strategy for several human malignancies (Xie et al., 2016). In general, inhibitors of the PI3K/AKT/mTOR network can be grouped as: 1) AKT blockers, 2) Pan-PI3K/mTOR blocker, 3) PI3K blocker, 4) Rapalogs (temsirolimus, everolimus, and deforolimus), and 5) mTOR blocker (Xie et al., 2016). The mTor pathway and checkpoints where it can be blocked are presented in Figure 4.

### Immunosuppressive Immune Cells in the TNBC Tumor Microenvironment

The tumor microenvironment (TME) involves the surrounding blood vessels, fibroblasts, immune cells, signaling molecules and the extracellular matrix around the tumor (Deepak et al., 2020). Tumor Infiltrating Lymphocytes (TILs) produce endogenous antitumor immune response for inhibiting tumor progression and improving free survival rate of TNBC patients (Reis-Filho and Tutt, 2008; Yu and Di, 2017). Tumor associated macrophages are important for immunosuppressive role by secreting inhibitory cytokines, regulatory T cells infiltration promotion, and reactive oxygen species reduction (Yu and Di, 2017). Cancer-Associated Fibroblasts lower anti-tumor immunity, favor tumor cell proliferation and invasion and reshape the extracellular matrix (Yu and Di, 2017). Tumor associated neutrophils aid in lysing tumor cells and induce antitumor function (Annaratone et al., 2020).

### Existing and Investigational Treatment Paradigm

From a chemotherapeutic perspective, TNBC is very sensitive, and treatments require extreme care. Common treatment involves the use of alkylating agents (such as cyclophosamide), anthracycline (doxorubicin topoisomerase blocker and DNA intercalating agents), anti-metabolite fluorouracil, and anti-microtubule agent (taxane) (Chang et al., 2019; Won and Spruck, 2020). For early diagnosis of TNBC, neoadjuvant chemotherapy and subsequent surgery are applied. No standard chemotherapy has been described for the treatment of relapsed TNBC. Treating advanced TNBC includes the following drugs: gemcitabine and capecitabine (anti-metabolites), eribulin (non-taxane microtubule inhibitor), and platinum (DNA cross-linker). The conventional treatment options for TNBC are listed in Table 1.

**TABLE 1:** Conventional treatment option for TNBC.

Treatment	TNBC Type	Drugs	References
Neo-adjuvant Therapy	Early TNBC, Advanced or Metastatic	Capecitabine + Taxane	Ali and Wendt, (2017)
		Anthracyclines + Taxanes	
		Capecitabine + Ixabepilone	
		/Ixabepilone monotherapy	
New Neo-adjuvant agents	BRCA mutations	Nab-paclitaxel, evacizumab, Carboplatin	Sikov et al. (2015)
Adjuvant agents	Early TNBC	Taxanes and Anthracyclines	Martin et al. (2013)

For advanced TNBC, new therapies have been reported, particularly when surgery is not desired. Compared to other breast cancer subtypes, TNBCs show greater immunogenicity. They have tumor-infiltrating lymphocytes in their microenvironment and express programmed cell death ligand (PD-L1) in high order (Stanton et al., 2016). Considering the therapeutic potential of the PD-L1 pathway, several immunotherapies have been explored, and atezolizumab combined with nanoparticle albumin-bound paclitaxel has been approved by the US FDA as the first-line therapy in 2019 (Schmid et al., 2018). Pembrolizumab was approved in 2017 as an anti-PD-1 antibody (Dudley et al., 2016). In 2018, talazoparib and olaparib were approved by the FDA for treating HER2 negative breast cancer (Won and Spruck, 2020). With the aim of improving TNBC treatment, several therapeutic strategies have been explored in clinical studies, including those that target or are immune specific for tumor stroma, intracellular or surface receptors, DNA damage response, and signaling pathways. So far, 399 studies have been shown on ClinicalTrials. gov for TNBC and are under phase III investigation (Ahmed et al., 2014; Riaz et al., 2017; Won and Spruck, 2020). The concept of immune checkpoint inhibitors is to halt regulatory immune checkpoints and thus activate anti-tumor responses. This treatment strategy is considered a game changer in cancer therapy and involves molecules that can negatively alter the immune response. Immune checkpoints can be readily blocked by antibodies or modified by recombinant ligands (Pardoll, 2012; Muneer et al., 2021). Immune checkpoint inhibitory antibodies aid in the attachment of molecules capable of stopping the immune response to tumor-infiltrating lymphocytes, leading to reactivation of antitumor immune responses (Singh et al., 2021). Research regarding the use of immune checkpoint inhibitor(s) either alone as a single agent or in combination therapy is ongoing (Adams et al., 2019). Neoadjuvant therapy has yielded mixed results. Promising anti-tumor activity and considerable safety were noted when neoadjuvant chemotherapy was used in combination with pembrolizumab in early stage TNBC (Nanda et al., 2020). In one study, neoadjuvant combination therapy revealed a higher pathologic complete response rate (65%) than the placebo-chemotherapy group (61%). In another study, neoadjuvant chemotherapy was investigated with or without atezolizumab for early stage and high-risk unilateral breast cancer. However, no improvement was seen in the pathologic complete response using the combination therapy (Gianni et al., 2019). Immunotherapy involving targeting of the 2B receptor (A_2b_R) and adenosine 2A receptor (A_2a_R) is considered a promising approach for the reactivation of antitumor immunity and enhancement of cytotoxic T cell immune responses (Ohta, 2016). In different clinical trials, the combination of immune checkpoint inhibitors and adenosine pathway inhibitors has been investigated. For example, NZV930, which is an anti-CD72 antibody when used alone or in combination with a PD-1 inhibitor or A_2a_R antagonist to block adenosine mediated inhibition of T lymphocytes. In another ongoing study, NIR178 was used in combination with spartalizumab for diffuse large B-cell lymphoma and multiple solid tumors to check whether the addition of an antagonist improves PD-1 inhibition efficacy. In addition, AB928 (a dual adenosine A_2a_R/A_2b_R receptor antagonist) is being evaluated in combination with AB122 (PD-1 inhibitor) for patients with advanced malignancies (Powderly et al., 2019). Different types of poly (ADP-ribose) polymerase inhibitors have also been described. These inhibitors include niraparib, veliparib, olaparib, talazoparib, and rucaparib (Won and Spruck, 2020). The role of androgen receptor inhibitors in TNBC still needs to be explored, and more insights need to be explored. The first-generation androgen receptor antagonist bicalutamide is a proof of concept for treating advanced TNBC, and the results showed a modest clinical benefit rate of 19% (Gucalp et al., 2013). Abiraterone, a second-generation anti-androgen inhibitor, shows promising targeting of androgen biosynthesis (Bonnefoi et al., 2016). Another second-generation androgen receptor inhibitor, enzalutamide, showed competitive binding to the androgen receptor ligand-binding domain and blocked its nuclear translocation, coactivator recruitment, and DNA binding (Traina et al., 2018). Seviteronel targets estrogen and androgen production (Gucalp et al., 2017). In addition to these therapeutic options, cell surface targets, such as tumor-associated carbohydrate antigens, have been explored as antigens for vaccine formulation. In particular, the Globo H antigen, which is expressed on the surface of different cancer types, can be explored for vaccine design. Adagloxad simolenin is an immunostimulating agent consisting of a Globo H hexasaccharide epitope fused with a keyhole limpet hemocyanin protein carrier (Gilewski et al., 2001). The antibody-drug conjugate remains stable in plasma, attacking antigens at the tumor cell surface with high specificity and affinity, followed by internalization, cleavage, and release of the payload drug to exert anti-tumor activity. For example, Sacituzumab govitecan-hziy targets human trophoblast cell-surface antigen 2, which is present in more than 90% of TNBCs. In this case, the active metabolite is irinotecan in conjugation with anti-trophoblast cell-surface antigen 2 antibody (Bardia et al., 2019). Ladiratuzumab vedotin or a short LV main target is a transmembrane protein (LIV-1). The protein has metalloproteinase and zinc transporter activity and is expressed in more than 90% of breast tumors. Ladiratuzumab vedotin comprises the microtubule-disrupting agent monomethyl auristatin E as payload (Bonnefoi et al., 2016). In addition to these therapeutic strategies against TNBCs, new platforms have been described. In this approach, EZH2 inhibitors were evaluated against the CDK2-EZH2 axis, thus reactivating ERα expression (Nie et al., 2019). In a recent study, researches combined the PARP inhibitor with CSF-1R blocking antibodies to elucidate the key contribution of immunosuppression to limiting the effective anti-tumor response their study demonstrates that combining the PARP inhibition with macrophage targeting therapy induces a durable reprograming of the tumor microenvironment and can be used as a promising therapeutic strategy for TNBC (Mehta et al., 2021). In another new technique, the combination of histone deacetylase and DNA methyltransferase inhibitor results in ERα expression in breast cancer models (Yang et al., 2001). The different types of nanomedicines under experimental and clinical testing for TNBC theranostics are tabulated in Table 2.

**TABLE 2:** Different types of nano medicines under experimental and clinical testing for TNBC theranostics (Medina et al., 2020).

Nanoparticle	Status	Therapeutic applications	References
Fluorescent nano-diamonds	Experimental and under clinical testing	It uses non-radioactive materials for imaging and has enhanced specificity and sensitivity	Fudala et al. (2013)
Quantum dots	Experimental and under clinical testing	Quantitative detection and cancer imaging	(Michalet et al., 2005; Zheng et al., 2016)
Silver nanoparticles	Experimental and under clinical testing	Cytotoxicity to cancer cells	Swanner et al. (2015)
Superparamagnetic iron oxide nanoparticles	Experimental and under clinical testing	Induce tumor apoptosis	Vyas et al. (2015)
Iron oxide nanoparticles	Experimental and under clinical testing	Produce contrasting images in MRI	Hayashi et al. (2013)
Gold nano-stars	Experimental and under clinical testing	Drug delivery, hyperthermia, theranosics and gene therapy	Zhang et al. (2021)
Core-shell nanoparticles	Experimental and under clinical testing	Generate apoptosis	Meng et al. (2018)
Nanocages	Experimental and under clinical testing	Hyperthermia, Immunotherapy, theranostics, and photodynamics	Liang et al. (2018)
Nanocomposites	Experimental and under clinical testing	Hyperthermia, Immunotherapy, theranostics, drug delivery and photodynamics	Liao et al. (2019)
Nanorods	Experimental and under clinical testing	Hyperthermia, Immunotherapy, gene therapy, theranostics, drug delivery and photodynamics	Feng et al. (2015)

### Targeting Tumor Microenvironment for TNBC Therapy

**FIGURE 5:**
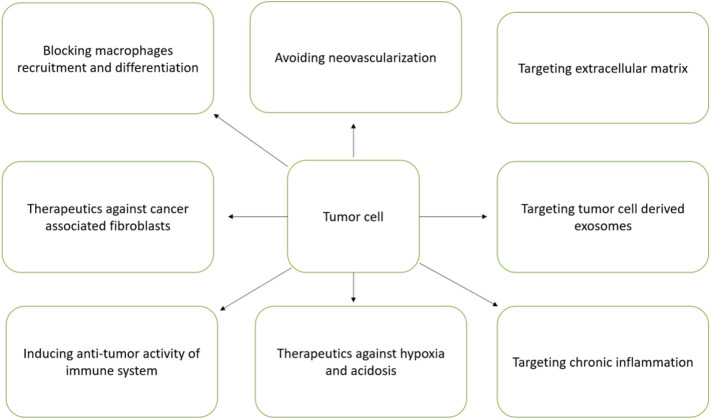
Different TME targets for therapeutic intervention.

The development of TNBC has strong association with the physiological state of TME. TNBC has been characterized with unique TME and is different from other subtypes (Roma-Rodrigues et al., 2019). TME has strong association with induction of angiogenesis, proliferation, apoptosis inhibition, suppression of immune system and resistance to drugs (Kuroda et al., 2021). The exosomes function as promising nanovesicles that directs TME orchestration by communicating cells within TME milieu (Deepak et al., 2020). The different components of TME particularly the soluble factors, transformed extracellular matrix, immune suppressive cells, re-programmed fibroblasts and epigenetic modifications altogether helps in TNBC progression and metastasis (Deepak et al., 2020). Hence, TME is regarded as a good therapeutic target. The different TME targets for therapeutic intervention is schematically presented in Figure 5.

### Prognosis of TNBC

The prognostic research of TBNC is mostly performed as retrospective studies, and these studies considered diagnostic data. Most of these studies used the triple negativity inclusion factor and neglected molecular markers for basal-like breast cancer. Poor prognosis has been observed in patients with TNBC (Nofech-Mozes et al., 2009). In contrast to other subtypes, TNBC development occurs more frequently in pre-menopausal women during early life. TNBC has a more aggressive expression profile (high p54 and Ki67 and low Bcl-2 expression), large tumor size, and high nuclear mitotic grade (Rhee et al., 2008). Many studies have demonstrated lower RFS in TNBC than in non-TNBC patients. The 4-year survival rate of TNBC patients was 85.5%, which is comparable to that of non-TNBC patients (94.2%) (Rhee et al., 2008). In another study, relapse frequency was less frequently reported in TNBC (Parikh et al., 2008). Tumor recurrence is 1.2 years which is shorter than that in non-TNBC patients. Similarly, TNBC has a worse prognosis in patients with recurrent breast cancers. The risk of tumor recurrence and death is high in TNBC, in contrast to the other types. It has been reported that the hazard ratio (HR 4.2 for developing TNBC tumor recurrence when compared to other cancers (Mersin et al., 2008). For triple-positive breast cancer, the 5 year survival is 91%, whereas for TNBC and HR-positive/HER2-negative cancers, it is 81 and 94%, respectively, (Kaplan and Malmgren, 2008).

### Artificial Intelligence in TNBC Management

In the last decade, the power of artificial intelligence in different scientific fields, particularly in medicine, has been seen as a real tool for the efficient diagnosis and management of diseases. Artificial intelligence-based applications in medical sciences include computer-aided detection and diagnosis of diseases, case-based reasoning, explainable artificial intelligence, osteodetect machine learning, and rainbox boxes (Wang et al., 2018). Decision Support and Information Management System for Breast Cancer (DESIREE) is a product of artificial intelligence that can be used in cancer prediction and interpretation of clinical data. Different artificial intelligence-based tools are also used for breast cancer screening. The metadata, which serve as the basis of these artificially based algorithm designs, are provided by the cancer data bank along with patient biodata history and treatment trends. The data are analyzed both quantitatively and qualitatively to train the algorithms so that the tools/servers can assist physicians in faster and more accurate ways to detect and manage complex cancer types. Recently, Fernández Martínez et al. reported a machine-learning algorithm that is capable of detecting different TNBC subtypes. The Cancer Genome Atlas (TCGA) is another platform that collects SNP data, DNA and RNA sequence data, and reserve phase protein array information. Together with the Molecular Taxonomy of the Breast Cancer International Consortium (METABRIC), TCGA aims to provide data related to molecular heterogeneity of different breast cancer subtypes. Both databases could help oncologists in advising personalized medicine for cancer patients in a timely manner. Very limited work has been done so far in this field, and much more interdisciplinary efforts are required to put solid platforms that cannot easily diagnose TNBC early, but at the same time allow efficient and effective treatment.

## Conclusion

Among all breast cancers, TNBC has the worst prognosis, in addition to suppressed immunity of the patient. Hence, understanding the TNBC molecular signaling pathway in the tumor microenvironment by applying multidisciplinary research will greatly advance TNBC diagnosis and therapy. In-depth information of TNBC pathways at the genetic and proteomic levels can assist in novel therapeutic design and successful trial development. New developments in computer biology and discoveries in immunology, nanotechnology, and molecular biology will ease earlier diagnosis and personalized treatment.
